# Derepression of the Plant Chromovirus *LORE1* Induces Germline Transposition in Regenerated Plants

**DOI:** 10.1371/journal.pgen.1000868

**Published:** 2010-03-05

**Authors:** Eigo Fukai, Yosuke Umehara, Shusei Sato, Makoto Endo, Hiroshi Kouchi, Makoto Hayashi, Jens Stougaard, Hirohiko Hirochika

**Affiliations:** 1 National Institute of Agrobiological Sciences, Tsukuba, Ibaraki, Japan; 2 Kazusa DNA Research Institute, Kisarazu, Chiba, Japan; 3 National Institute of Crop Science, Tsukuba, Ibaraki, Japan; 4 Centre for Carbohydrate Recognition and Signalling, Department of Molecular Biology, University of Aarhus, Aarhus, Denmark; Iowa State University, United States of America

## Abstract

Transposable elements represent a large proportion of the eukaryotic genomes. Long Terminal Repeat (LTR) retrotransposons are very abundant and constitute the predominant family of transposable elements in plants. Recent studies have identified chromoviruses to be a widely distributed lineage of *Gypsy* elements. These elements contain chromodomains in their integrases, which suggests a preference for insertion into heterochromatin. In turn, this preference might have contributed to the patterning of heterochromatin observed in host genomes. Despite their potential importance for our understanding of plant genome dynamics and evolution, the regulatory mechanisms governing the behavior of chromoviruses and their activities remain largely uncharacterized. Here, we report a detailed analysis of the spatio-temporal activity of a plant chromovirus in the endogenous host. We examined *LORE1*a, a member of the endogenous chromovirus *LORE1* family from the model legume *Lotus japonicus*. We found that this chromovirus is stochastically de-repressed in plant populations regenerated from de-differentiated cells and that *LORE1*a transposes in the male germline. Bisulfite sequencing of the 5′ LTR and its surrounding region suggests that tissue culture induces a loss of epigenetic silencing of *LORE1*a. Since LTR promoter activity is pollen specific, as shown by the analysis of transgenic plants containing an LTR::GUS fusion, we conclude that male germline-specific *LORE1*a transposition in pollen grains is controlled transcriptionally by its own *cis*-elements. New insertion sites of *LORE1*a copies were frequently found in genic regions and show no strong insertional preferences. These distinctive novel features of *LORE1* indicate that this chromovirus has considerable potential for generating genetic and epigenetic diversity in the host plant population. Our results also define conditions for the use of *LORE1*a as a genetic tool.

## Author Summary

In contrast to animals, where germline differentiation initiates early in embryogenesis, germline differentiation in plants starts in the adult phase during reproductive development. Transpositions of transposable elements in both somatic and gametic cells can be transmitted to the next generation. As a result, plant genomes may contain transposable elements exhibiting a variety of tissue-specific activities. Thus far, the spatio-temporal activity of LTR retrotransposons, the most abundant class of transposable elements in plants, has not been well characterized. Here, we report a detailed analysis of the spatio-temporal transposition pattern of a plant LTR retrotransposon in the endogenous system. Using the model legume *Lotus japonicus*, we found that *LORE1*a, a member of the chromovirus *LORE1* family that belongs to the *Gypsy* superfamily, was epigenetically de-repressed via tissue culture. Activation was stochastic and derepression was maintained in regenerated plants. This feature made it possible to trace the original spatio-temporal activity of the retrotransposon in the intact plants. We determined that the plant chromovirus retrotransposes mainly in the male germline, without obvious insertional preferences for chromosomal regions. This finding suggests that the tissue specificity of transposable elements should be taken into account when considering their impact on the host genome dynamics and evolution.

## Introduction

A large proportion of the eukaryotic genome is composed of transposable elements (TEs). In flowering plants, Long Terminal Repeat (LTR) retrotransposons have been regarded as the largest order of TEs [1],[2] and it has been suggested that the ratio between propagation and exclusion of LTR retrotransposons may have affected the size of host genomes [3],[4]. In line with this notion, large plant genomes usually contain substantially more LTR retrotransposons than small plant genomes [5],[6]. However, data from a wide range of flowering plants strongly suggest that LTR retrotransposons are not distributed evenly in genomes. Biased accumulation has led to the formation of LTR retrotransposon-rich, gene-poor heterochromatic blocks, which separate gene-rich euchromatic regions [7]. Thus, the activity of LTR retrotransposons has contributed remarkably towards generating the basic structure of current plant genomes.

In flowering plants, the LTR retrotransposons have been classified into two superfamilies, *Gypsy* and *Copia*, according to their structural features [8]. In many plants, *Gypsy* outnumbers *Copia* [9]–[13]. An exception is grapevine, in which the number of *Copia* elements exceeds that of *Gypsy* [14]. Chromovirus is a most widely-distributed lineage of *Gypsy*, characterized by a chromodomain at the carboxyl terminal of the ORF [15],[16]. It has been proposed that the insertion site preference of chromoviruses is controlled by the chromodomain [15],[16], and this suggestion has been supported by functional characterization of *MAGGY*, identified in the rice blast fungus *Magnaporta grisea* [17],[18]. The *MAGGY* chromodomain was shown to interact with histone H3 di- and tri-methyl K9, which are hallmarks of heterochromatin [18]. When it was fused to the integrase of Tf1 retrotransposon, the modified Tf1 preferentially transposed into heterochromatic regions in *Schizosaccharomyces pombe* genome [18]. In flowering plants, chromoviruses are phylogenetically distinct from the lineage containing *MAGGY* and they are classified into four clades, Reina, Tekay, Galadriel and CRM [16],[19]. Members of CRM were originally known as *Gypsy* elements which accumulate in centromeric and pericentromeric regions in plant genomes [20]–[23]. Since all four clades have been identified in both dicots and monocots, and Reina and CRM elements have been found in angiosperms and gymnosperms, these elements are likely to have an ancient origin within the seed plants [16],[19]. In order to complement these evolutionary studies, a precise characterization of retrotransposon transpositional activity is now being pursued by experimental analyses, and this activity represents one of the subjects that must be addressed if we are to develop a deeper understanding of plant genome dynamics and evolution.

Previously, most experimental studies of transpositional activity and the regulation of plant LTR retrotransposons were conducted using three *Copia* elements, *Tnt1* and *Tto1* in tobacco, and *Tos17* in rice. Transpositions of these elements were observed only in cultured cells, where their transcriptional up-regulation occurs [24]–[26]. Since transpositional activity is immediately repressed in regenerated plants due to a decrease in transcription, transpositions in intact plants have not been well characterized. Thus far, transposition of *Tos17* has been observed in intact transgenic plants in which the transcriptional level of a gene encoding histone H3K9 specific methylase was downregulated by RNA interference [27], but the spatio-temporal pattern of transposition remained unclear. Furthermore, little is known about the transpositional activity of plant *Gypsy* elements, including chromoviruses, despite their high abundance in plant genomes.

**Figure 1:**
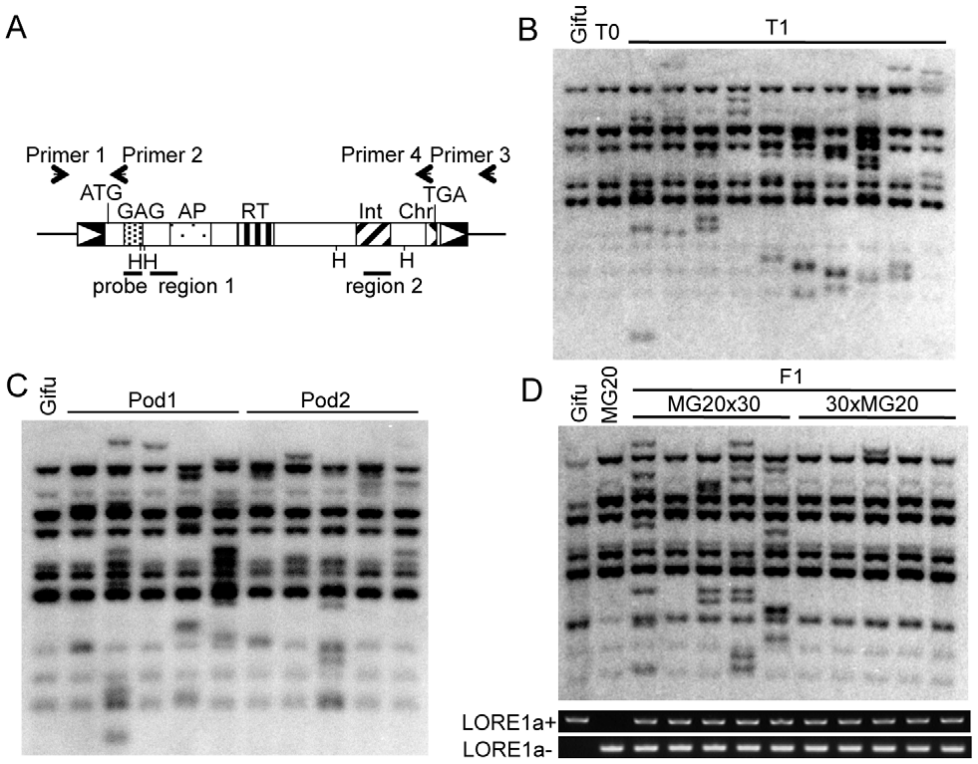
*LORE1* transposition. (A) Schematic representation of the *LORE1*a element. Boxes with triangles represent long terminal repeats (LTRs). Regions encoding functional domains predicted by Pfam are indicated as patterned boxes. GAG, Retrotransposon gag protein; AP, Aspartic protease; RT, reverse transcriptase; Int, integrase core domain; Chr, chromodomain. Positions of Primers 1, 2, 3, and 4 are indicated with arrows. Regions 1 and 2, containing *LORE1*a-specific SNPs, are indicated as bars. Positions of the DNA probes and *Hin*d III sites used in the genomic Southern blot analyses shown in (B–D) are represented as bars and vertical lines with H, respectively. (B) Southern blot detection of transposed *LORE1* elements in T1 siblings. *LORE1* copies in control Gifu (Gifu), a T0 individual (T0), and 10 of its T1 siblings (T1) were analyzed. (C) *LORE1* transposition in late development. Five siblings originating from each of two pods, pod1 and pod2, set at the top of the same inflorescence of a *LORE1*-activated T0 plant. (D) Southern blot analysis showing germline inheritance of *LORE1* via male gametophytes. DNA from five F1 plants from each of the MG20 (female) × no. 30 (male) and no. 30 (female) × MG20 (male) crosses was analyzed. Gel images show PCR products obtained using Primers 1 and 2 detecting *LORE1*a (+*LORE1*a) from Gifu, and Primers 1 and 3 detecting absence of *LORE1*a (−*LORE1*a), the allele from MG20. Amplification of both bands confirmed the hybrid genotype of F1 plants.

In more than a decade of studies, the model legume *Lotus japonicus* has facilitated dissection of the molecular mechanisms governing symbiotic nitrogen fixation with rhizobia. The *L. japonicus* genome has been sequenced and sequence data covering 67% of the genome (472 Mb), corresponding to 91.3% of the gene space, is now available [13]. From this model legume, we have identified two transpositionally active LTR retrotransposon families designated as *LORE1* and *LORE2* (*Lotus Retrotransposon 1* and *2*) [28],[29]. Both belong to the *Gypsy* superfamily and were first identified as insertions in symbiotic mutants isolated from a transgenic plant population established by tissue culture- mediated transformation [28]–[31]. However, the machinery underlying their activation remained to be characterized. Both *LORE1* and *LORE2* encode unique long open reading frames (ORFs) with a chromodomain at the carboxyl terminal ends, which suggests that they are chromoviruses (Figure 1A) [29]. Although this chromodomain was overlooked in the original characterization of *LORE1* [28], Novikova *et al.* re-classified *LORE1* as a member of the Reina clade of chromovirus [19].

Previously, we estimated the number of “preexisting copies” (insertions that were already present in a plant accession) of *LORE1* in the Gifu accession as ten, and obtained full or partial sequences for nine out of the ten preexisting *LORE1* copies [28]. Nucleotide sequence polymorphisms among the nine copies enabled us to distinguish them from each other, and we designated them in alphabetical order as *LORE1*a, b, c, d, e, f, g, h, and i [28]. In this report, we show that in the Gifu accession, the preexisting *LORE1*a can be epigenetically de-repressed in standard tissue culture. However, transpositions per se occur primarily in pollen, i.e., male gametophytes, of regenerated intact plants, and so far new insertions generated in cultured cells have not been detected. We assume that the pollen-specific LTR promoter of *LORE1*a regulates the spatio-temporal pattern of transposition. Although *LORE1* is a chromovirus, it does not appear to have a strong insertional preference for heterochromatin. These distinctive features of *LORE1* underlie its ability to generate insertional polymorphisms, leading to a wide range of genetic and epigenetic diversity in a population. The results also define conditions for using *LORE1* for insertion mutagenesis.

## Results

### Activation of *LORE1* in regenerated plant populations

The transpositional activity of *LORE1* was first demonstrated by the identification of four symbiotic mutant alleles, *nin-7*, *symrk-1*, *nup133-3* and *nap1-1*, in which gene inactivation was caused by the insertion of *LORE1* [28],[32]. As all four mutants were isolated from the same *Ac*/T-DNA tagging population established using the *L. japonicus* Gifu accession [30],[31], we screened other plants of the same population for *LORE1* transpositions. Sequence-specific amplified polymorphism (SSAP) analysis of *LORE1* insertion sites detected new transpositions in 32 plants out of a sub-population of 41 plants (Population 1 in Table 1), indicating that *LORE1* was widely active in this population. Next, we investigated whether *LORE1* transpositions were present in four transgenic or non-transgenic regenerated plant populations created using the Gifu accession. To detect new insertion sites of *LORE1*, we used SSAP to analyze the T1 and R1 progeny of primary transformants (T0) and of primary non-transgenic regenerated plants (R0). In addition to population 1 (Table 1), transpositions were detected in three of the other four populations. Importantly, transposition was detected in transgenic plants generated using six different constructs, as well as in non-transgenic regenerated plants. These results suggest that the simple process of *in vitro* tissue culture can activate *LORE1* in a stochastic manner that is independent of the presence or absence of transgenes, antibiotic selection, and of the composition and contents of transgene constructs. The newly transposed *LORE1* copies observed in R1/T1 plants might have resulted from transpositions in cultured cells and/or in the parental R0/T0 plants. However, *LORE1* transposition was absent, infrequent, or below the detection levels of the SSAP method in a total of 27 plants from the initial R0/T0 plants from populations 2 and 3 (Table 1; data not shown). Previously, we observed the absence of obvious transcriptional or transpositional activation of *LORE1* in cultured cells [28],[29]. These results suggest that even though *LORE1* was apparently de-repressed in tissue culture, the transpositions per se appear to have occurred in regenerated intact plants, rather than in the cultured cells (see details in the next section).

**Table 1:** *LORE1* transpositions in regenerated plant populations.

Population	Generation investigated	No. of plants investigated	No. of plants with *LORE1* transpositions#	Origin	Antibiotics used
1. Ac/T-DNA tagging population generated in Denmark	Later than T2	41	32	-	G418
2. Regenerated plants produced in Japan	R1	50	10	10 R0 plants	None
3. Transgenic plants generated in Japan	T1	88	42	17 T0 plants	Hygromycin or G418
4. Other transgenic plants generated in Denmark	T1	31	1	6 T0 plants	G418
5. Regenerated plants produced in Japan	R1	45	0	9 R0 plants	None

**#**Transpositions were detected by SSAP (Sequence Specific Amplified Polymorphism) analysis amplifying 5′ fragments flanking *LORE1* inserts.

### Transposition of *LORE1* in intact plants

To gain more precise information about transposition of *LORE1* in intact plants, eight independent T0 plants were randomly selected from population 3 (Table 1) and investigated together with their T1 progeny. *LORE1* transpositions were detected in the T1 progeny from 6 of the 8 T0 plants. A typical result of a genomic Southern blot analysis and SSAP analysis of a T0 and its 10 T1 progeny plants (in this instance plant line no. 30) are shown in Figure 1B and Figure S1, respectively. Notably, the banding pattern in the T0 plants was the same as in the control Gifu, again indicating absent or infrequent *LORE1* transposition in the primary regenerated plants (Figure 1B). However, additional bands corresponding to newly transposed *LORE1* copies were detected in the T1 progeny (Figure 1B and Figure S1). The highly polymorphic banding pattern indicates the occurrence of frequent independent transpositions of *LORE1* in T1 plants (Figure 1B and Figure S1).

Next, we determined whether the new insertions of *LORE1* found in the T1 plants were the result of transmission of previous transpositions in somatic cells from T0 forming sectors or of *de novo* transposition. We analyzed T1 plants originating from two seed pods at the top of the same shoot of the parental T0 plant (Figure 1C). We did not detect any new bands that were shared by the two neighboring pods, or T1 plants originating from the same pod. This result indicates that the majority of *LORE1* transpositions occurred at late developmental stages in T0 plants. Reciprocal crosses between plant no. 30 (from the Gifu accession) and plants from the MG20 accession were used to determine if the new transposed copies detected in the T1 plant were transmitted via male or female gametes. Five F1 plants obtained from each reciprocal cross were analyzed for *LORE1* copy number (Figure 1D). In total, 21 bands corresponding to new *LORE1* transpositions were detected among the 5 F1 plants obtained from the MG20 (female) × no. 30 (male) cross. In contrast, only 1 newly transposed *LORE1* copy was detected in 5 F1 plants from no. 30 (female) × MG20 (male) cross. We conclude that although *LORE1* is active in both male and female gametophytes, its activity is much higher in male tissues. Next, we used parent-specific single nucleotide polymorphisms (SNPs) in the flanking regions to determine the parental origin of the seven new insertion sites in MG20x30 F1 plants. This analysis showed that all the new transpositions originated from Gifu, the pollen donor. Hence, the majority of *LORE1* transpositions detected in the F1 plants seemed to occur before fertilization. Altogether, *LORE1* was revealed to be robustly active especially in male gametophytes. Previous reports indicate that activated retrotransposons can be re-silenced again by activities such as copy number-dependent establishment of epigenetic silencing [33],[34]. However, *LORE1* was still active in three T1 plants that already possessed an increased number of *LORE1* copies (Figure S2A). This finding indicates that once activated, *LORE1* was able to transpose over at least two successive generations. On the other hand, we also observed that *LORE1* was inactivated in the *nup133-3* mutant, in which a single new transposition was detected in the *Nup133* gene (Figure S2B).

### Activation of *LORE1*a in the initial generation of regenerated plants

Since the newly inserted *LORE1* copies identified in the three symbiotic mutant alleles (*nfr5-2*, *symrk-2*, and *nup133-3*) were identical to one of the nine preexisting copies, *LORE1*a, we suspected that *LORE1*a was preferentially activated [28]. *LORE1*a-specific SNPs were identified in regions 1 and 2 (Figure 1A) and in all eight of the newly-transposed *LORE1* fragments from population 3. This observation is consistent with our suggestion that *LORE1*a is responsible for the majority of *LORE1* transpositions described here.

**Figure 2:**
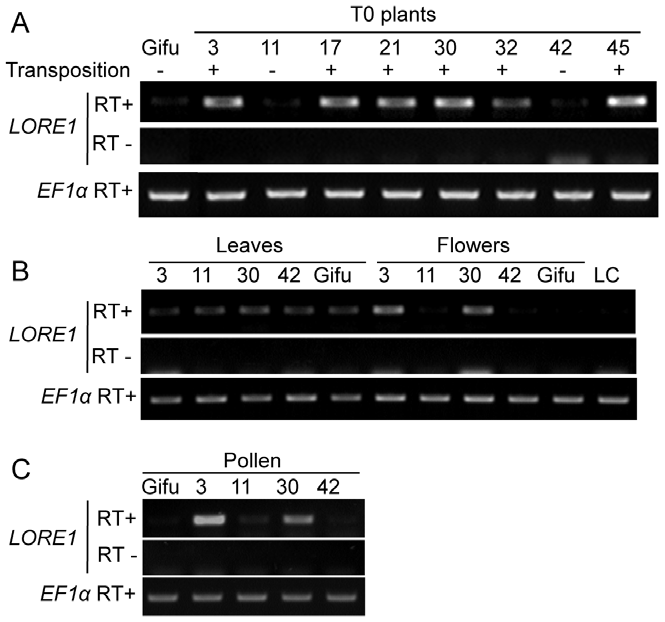
Correlation between transcription level and transpositional activity of *LORE1*. (A) *LORE1* transcript levels in T0 plant nos. 3, 11, 17, 21, 30, 32, 42, and 45, as well as in a control Gifu plant. Plants marked with + show transpositional activity of *LORE1*, those marked with–do not. The upper panel (RT+) shows an image of an RT-PCR of *LORE1* transcripts in flowers. The middle panel (RT−) shows negative control reactions without reverse transcriptase. The equal abundance of RNA among samples was confirmed by RT–PCR detection of elongation factor 1 alpha transcripts (*EF1α*). (B) *LORE1* transcript levels among T0 plants vary in flowers, but not in leaves. The levels of *LORE1* transcripts in flowers and leaves were determined by RT–PCR as in (A) using RNA samples extracted from four T0 plants, two with *LORE1* activity (nos. 3 and 30) and two without *LORE1* activity (nos. 11 and 42), together with a control Gifu plant and liquid-cultured cells (LC) from a Gifu plant. Images from a negative control experiment (RT−) and a control, to demonstrate equal abundance of RNA (*EF1α*), are also presented. (C) Transcriptional activation of *LORE1* in pollen. Transcript levels in mature pollen grains isolated from *LORE1*-activated T0 plants (3 and 30), T0 plants without *LORE1* activity (11 and 42), and control Gifu plants, were compared by RT–PCR. Images of a negative control experiment (RT−) and a control, to demonstrate equal abundance of RNA (*EF1α*), are also shown.

The transpositional activity of retrotransposons is often controlled at the transcriptional level [1], [2], [24]–[26]. We used RT-PCR to compare the levels of *LORE1* transcription in mature flowers containing both male and female gametophytes (where *LORE1* transposition presumably occurs). Among the eight T0 plants, including no. 30 from population 3 (Table 1), higher levels of *LORE1* transcription were observed in the six T0 plants that possessed active *LORE1* elements, compared to the control Gifu plant (Figure 2A). This finding indicated a correlation between the transcriptional and transpositional activities of *LORE1*. To determine which *LORE1* family members were present in the transcript pool, RT-PCR products were TA cloned and sequenced. RT-PCR products spanning regions 1 and 2 were amplified separately from flowers of the control Gifu plant and two T0 plants (nos. 30 and 45) that exhibited *LORE1* activity. *LORE1*a-specific SNPs were present in the region 1 of 7/16, 15/15 and 15/16 clones from the control Gifu, no. 30 and no. 45 plants, respectively. For region 2, *LORE1*a-specific SNPs were present in 3/12, 16/16 and 16/16 clones from the control Gifu, no. 30 and 45 plants, respectively. These data suggest that transcriptional activation is responsible for the preferential transposition of *LORE1*a among the family members. This expectation is supported by the following lines of evidence: i) a generally increased level of *LORE1* transcripts in flowers of active lines; ii) a clear increase in *LORE1*a transcripts in two activated plants; and iii) all transposition events detected thus far are of *LORE1*a origin.

### Tissue specificity of *LORE1* activation

The pattern of *LORE1*a activation via tissue culture is different from that of other well-characterized retrotransposons such as *Tos17, Tto1*, and *Tnt1*, which are activated and transpose during tissue culture, resulting in a copy number increase in the primary regenerated plants (R0) [24]–[26]. We hypothesized that tissue- or cell-specific transcription determines the unique spatio-temporal pattern of *LORE1* transposition. To test this hypothesis, we compared *LORE1* transcript levels in leaves and flowers among four T0 plants and a control Gifu plant, as well as its transcriptional level in cultured cells (Figure 2B). We found that there were no detectable differences in *LORE1* transcript levels in the leaves of the four T0 plants or in the control Gifu plant. In contrast, high levels of *LORE1* transcripts accumulated in the flowers of plant nos. 3 and 30 compared to nos. 11 and 42, or the control Gifu plants and cultured cells. Furthermore, high *LORE1* transcript levels were detected in pollen from the two T0 plants exhibiting *LORE1* activity, compared to the two T0 plants without *LORE1* activity or the control Gifu plant (Figure 2C). These observations suggest that *LORE1* has transpositional activity in pollen and that tissue specificity is controlled at the transcriptional level.

**Figure 3:**
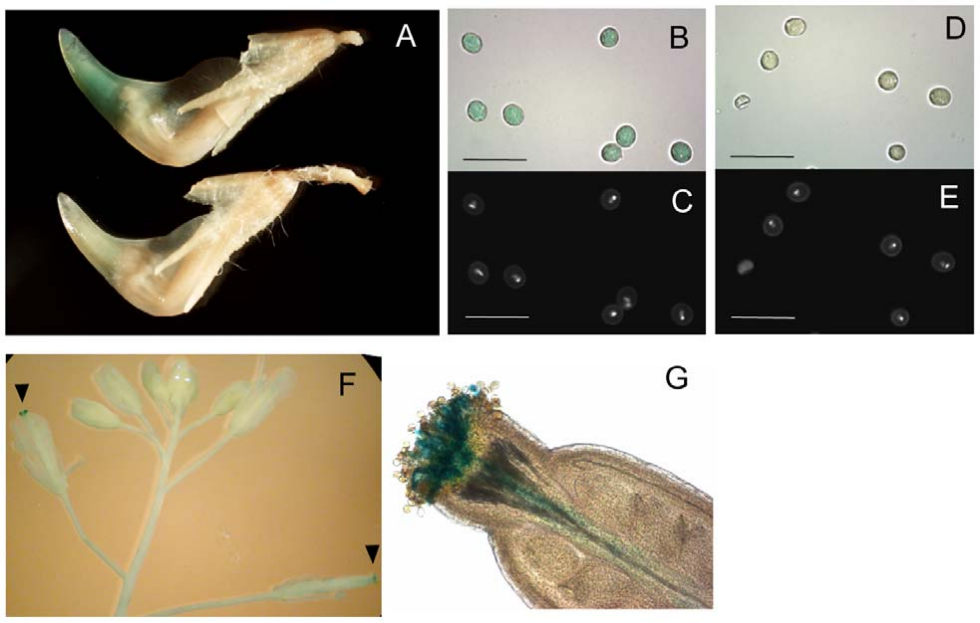
Promoter activity of the *LORE1*a LTR. Histochemical GUS assay of transgenic plants containing a *LORE1a* LTR::GUS fusion. (A) GUS staining was observed at the tip of keel containing released pollen grains in a transgenic *L. japonicus* plant (upper), while no GUS activity was detected in the control Gifu (lower). (B,D) Close up of pollen grains after GUS staining of transgenic (B) and control Gifu (D) samples. Scale bars correspond to 50 µm. (C,E) Hoechst 33258 staining of the pollen grains shown in (B,D), respectively. (F) A transgenic *Arabidopsis* inflorescence incubated with GUS substrate for 12 h. Blue staining (GUS positive) was observed only at the pollinated pistils, indicated with arrowheads. GUS activity was not detected in other tissues. (G) Magnified image of a GUS-positive stigma. GUS activity was observed in pollen tubes penetrating the stigma and in hydrated pollen on the stigma.

Since the 5′ LTR is known to function as a promoter for LTR retrotransposons [1], we determined promoter activity of the *LORE1*a LTR using a transgenic *L. japonicus* Gifu accession carrying *LORE1*a LTR fused to a GUS reporter gene. GUS activity was detected in mature pollen grains that were released from anthers and had accumulated at the tip on the inside of the keel (Figure 3A), as well as in isolated pollen grains (Figure 3B). We could not detect LTR-driven GUS activity in any other tissues (data not shown). A similar pattern of GUS activity was observed in three out of six independent transgenic plant lines. These results are in good agreement with the RT-PCR analyses, which indicate up-regulation of *LORE1* transcription in pollen grains (Figure 2C). To investigate the LTR promoter activity in a heterologous system, we generated transgenic *Arabidopsis* plants carrying the same construct. Four out of the seven *Arabidopsis* transgenic lines showed GUS activity in hydrated pollen grains on stigmas and in pollen tubes (Figure 3F and 3G). Prolonged staining for GUS activity detected weaker expression in developing young anthers (Figure S3A). In the youngest anthers showing activity, GUS was detected primarily in cell layers around the developing pollen, rather than in the developing pollen grains (Figure S3C and S3E). No GUS activity was detected in other tissues. Taken together, these results indicate that the *LORE1* LTR specifically promotes transcription in pollen and that the tissue specificity of the cis-elements may be operational in a wide range of flowering plants.

### Distribution of newly-transposed *LORE1* copies

**Figure 4:**
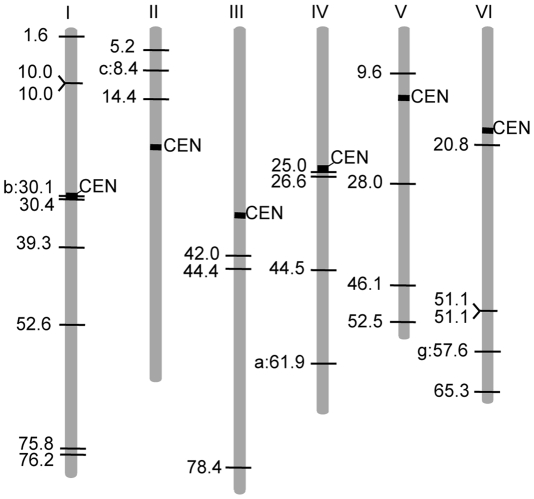
Linkage map positions of *LORE1* insertion sites. The Gifu map positions of 24 new insertion sites, together with the 4 preexisting *LORE1* elements. CM positions are indicated. Vertical bars with numbers indicate chromosomes. Lengths of chromosomes are represented in proportion to their genetic distances (http://www.kazusa.or.jp/lotus/). Regions predicted as centromeric and pericentromeric are indicated as black boxes (S. S. unpublished data). New insertion sites are indicated with horizontal lines. Positions of the four preexisting *LORE1* elements are indicated as horizontal lines with a letter identifying the individual copy.

The reported locations of several chromoviruses in the host plant genomes suggest that chromoviruses preferentially accumulate in heterochromatic regions [18], [20]–[23]. Of the nine preexisting *LORE1* copies so far identified, the insertion sites of *LORE1*d, e, f, h, and i were found in genomic clones containing highly repetitive sequences, which were potential heterochromatic regions. However, the remaining four, *LORE1*a, b, c and g, were found in contigs that did not display any apparent heterochromatic characteristics (S. S. unpublished data). To investigate whether *LORE1* exhibits a strong insertion site preference for heterochromatic regions, we used SSAP to obtain flanking sequences located immediately 5′ of new insertions in the T1 and R1 populations. A total of 97 SSAP fragments longer than 40 bp were analyzed by homology search using public databases including the *L. japonicus* genome sequence data obtained from the MG20 accession [13]. The absence of the 97 *LORE1* insertions in the wild-type Gifu accession was confirmed by PCR (data not shown). In this analysis, only sequences showing homology higher than 77%, along stretches longer than 40 bp and with bit scores larger than 58, were considered homologous sequences. For the 75% of the *LORE1* flanking sequences (73 out of the 97), homologous sequences including possible identical (allelic) sequences were identified from the published *L. japonicus* genome sequences (Table 2). The percentage (75%) is close to the coverage of the whole genome reported for the genome sequence project (67%) [13]. Among the 73 sequences, 37 were protein coding cellular genes or expressed sequence tags (ESTs), 11 were homologous to transposable elements (TEs), and the residual 25 did not show homology to genes or TEs and were categorized as unknown (Table 2). On the other hand, among the 24 fragments that did not show significant homology with *L. japonicus* sequences, 6 were classified as genes or ESTs, one was categorized as a TE, and the remaining 17 were classified as unknown (Table 2). Thus, a total of 43 sequences were assumed to be in genic regions. Among the 43, 31 were predicted to be exonic, since the insertion site was positioned in a region homologous to protein coding sequences and/or deposited ESTs. In contrast, 12% of the 97 *LORE1* flanking sequences showed homology with TEs, which is lower than the predicted TE content of the *L. japonicus* genome (36%) derived from end-sequencing data of randomly selected BAC clones (S. S. unpublished data). Finally, we physically mapped 24 of the 73 SSAP sequences, and 4 of the 9 preexisting *LORE1* members whose positions could be uniquely assigned, to the latest version of *L. japonicus* chromosome pseudo molecule [13] (Figure 4). This mapping indicated that the new insertion sites were distributed across the *Lotus* genome and no strong preference for *LORE1* insertion sites was observed from those data.

**Table 2:** Flanking sequences of new *LORE1* insertions.

Homologues in *L. japonicus* genome sequence data	Number (%)	Category	Number (%)
Yes	73 (75)	Genes or ESTs	37 (38)
		TE or repetitive	11 (11)
		Unknown	25 (26)
No	24 (25)	Genes or EST	6 (6)
		TE or repetitive	1 (1)
		Unknown	17 (18)
total	97 (100)		97 (100)

### Variation in cytosine methylation patterns at *LORE1*a among regenerated plants

**Figure 5:**
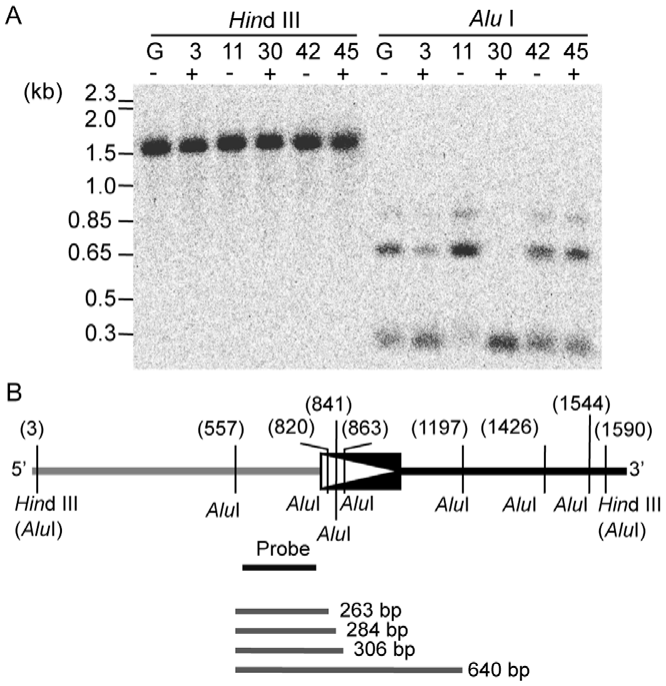
Variation in cytosine methylation status observed at three *Alu* I sites in the 5′ LTR of *LORE1*a from five T0 plants. (A) Genomic Southern blot detecting fragments containing 5′ DNA flanking of *LORE1*a. Genomic DNA samples of the Gifu control (G) and five T0 plants (nos. 3, 11, 30, 42, and 45) were extracted from leaves. DNA was digested with *Hin*d III (left) and *Alu* I (right). Plants marked with + show transpositional activity of *LORE1*, those marked with–do not. Molecular sizes of the DNA makers and the bands detected are indicated on the left. (B) A restriction map of the region around the 5′ LTR of *LORE1*a. 5′ LTR of *LORE1*a is indicated as a box with a triangle. The 5′ flanking region of *LORE1*a is indicated as a gray line on the left side of the LTR. The internal sequence of *LORE1*a is indicated as a black line on the right of the LTR. Two *Hin*d III sites closest to the LTR also contain *Alu* I sites. The seven *Alu* I sites are shown as vertical lines. Numbers in parentheses indicate the relative positions of the restriction sites in base pairs. The probe is shown as a black bar below the schematic of the genome region. Four *Alu* I fragments expected to correspond to the bands detected in the *Alu* I-digested genome samples are indicated as grey lines below.

Because of the frequent but stochastic derepression of *LORE1*a in regenerated plant populations (Table 1), we predicted that *LORE1*a activation accompanying tissue culture was induced epigenetically rather than genetically. We examined the status of cytosine methylation around the 5′ end of *LORE1*a by Southern blot analysis using two restriction enzymes, *Hin*d III and *Alu* I, which are sensitive to cytosine methylation at residues inside their recognition site [35]. We examined genomic DNA from five T0 plants (nos. 3, 11, 30, 42 and 45), together with the control Gifu (Figure 5A). When *Hin*d III was used to digest genomic DNA from leaves, we observed distinct bands (approximately 1.5 kb) in all of the five plants, suggesting the absence of cytosine methylation at the two *Hin*d III sites surrounding the region complementary to the DNA probe used in this analysis (Figure 5B). When genomic DNA samples were digested with *Alu* I, signals corresponding to approximately 300 and 650 bp DNA fragments were detected in each plant (Figure 5A). We assumed that the lower band signals represented a mixture of three *Alu* I fragments of 263, 284, and 306 bp, resulting from the digestion of the *Alu* I site 5′ adjacent to *LORE1*a and one of three *Alu* I sites in the 5′ LTR (Figure 5B). Thus, detection of the smaller hybridizing bands indicates the presence of hypomethylated *Alu* I sites in the 5′ LTR. On the other hand, the larger band was assumed to correspond to the 640 bp *Alu* I fragment, resulting from the absence of hypomethylated cleavable *Alu* I sites in the 5′ LTR (Figure 5B). Detection of signals from both high and lower sized DNA fragments indicates heterogeneity of the methylation status at the three *Alu* I sites in the 5′ LTR of each of the six investigated plants. However, the relative signal intensity of these DNA fragments showed variation among the five plants. The intensity of lower bands (corresponding to a hypomethylated status) was predominant in plant nos. 3 and 30, which have active *LORE1*a. However, the higher band (corresponding to hypermethylated alleles) was more intense in plant no. 11, which did not have active *LORE1*a. In plants nos. 42 and 45, both higher and lower bands were detected, with intensities similar to that of the control Gifu (Figure 5A). These trends in the relative signal intensity between the large and smaller sized bands were reproducible in independently extracted genomic DNA (Figure S4). The banding patterns observed in flowers, where transcriptional activation of *LORE1*a was observed, were similar to those observed in leaves (Figure S4). This finding suggests that no obvious changes in cytosine methylation pattern can be correlated to changes in *LORE1* transcriptional level between the two tissues. Altogether, it would appear that T0 plants have a variable epigenetic status for *LORE1*a, and that it is different from Gifu control plants.

**Figure 6:**
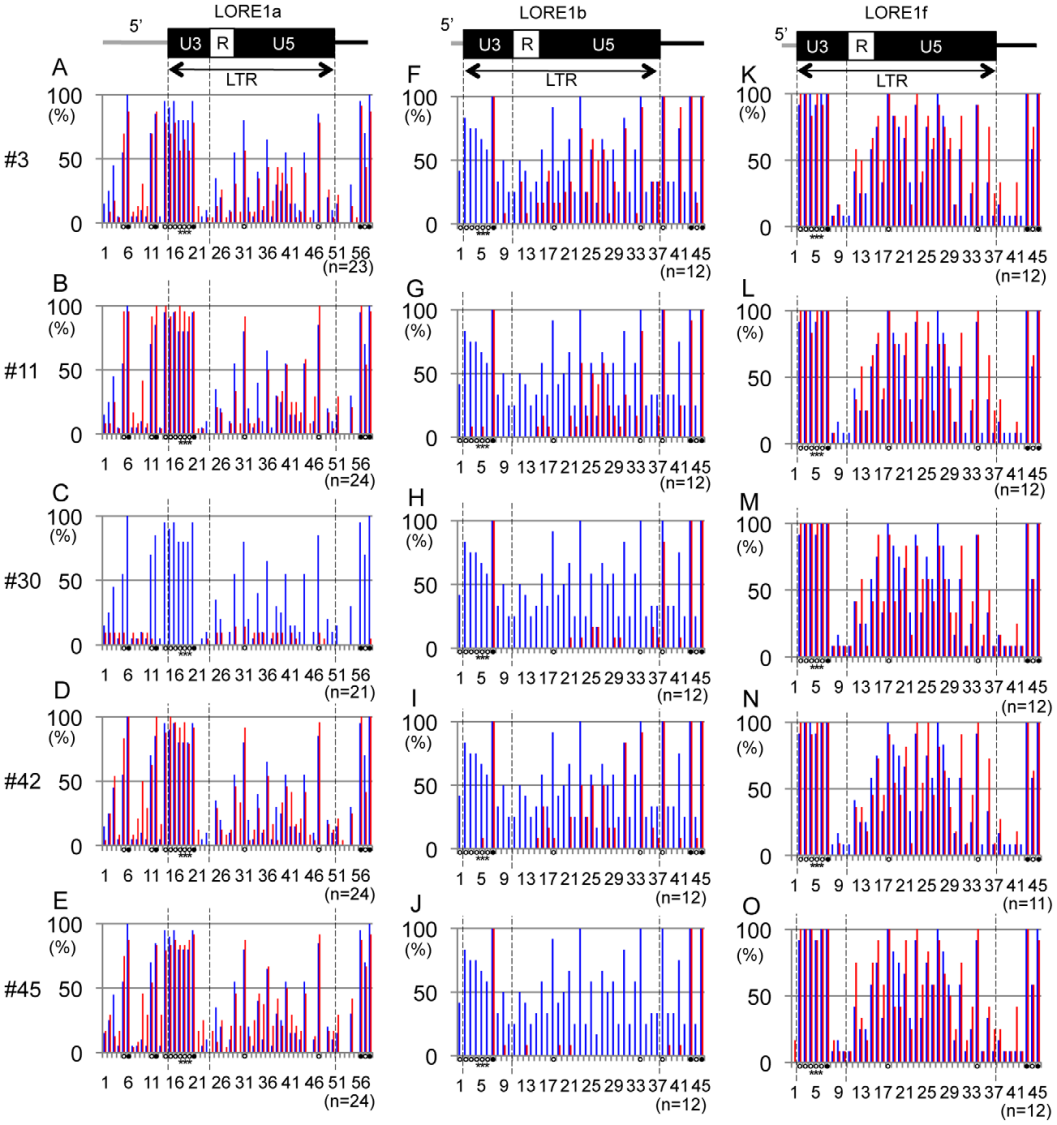
Cytosine methylation status around the 5′ LTR of *LORE1*a, *LORE1*b, and *LORE1*f. Bar graphs showing percentage of methylation at 58, 45, and 45 cytosines in the genomic regions around the 5′ LTR of *LORE1*a (A–E), *LORE1*b (F–J), and *LORE1*f (K–O), respectively. Methylation status in the Gifu control is represented by blue bars, while red bars indicate status in T0 plants. The positions of the 5′ flanking region, the 5′ LTR, and a part of the internal region of each *LORE1* locus were analyzed and are shown at the top. Regions correspond to LTRs and U3 regions are indicated with dotted lines. Closed and open circles correspond to the cytosines in CG and CHG contexts, respectively. Cytosines without any marks are present as CHH. Asterisks indicate cytosines in *Alu* I sites in the LTRs. The numbers of the sequenced TA clones are indicated at the bottom right of each bar graph.

An independent determination of the cytosine methylation status was obtained by bisulfite sequencing of the 5′ LTR of *LORE1*a in the five T0 plants and control Gifu. The same genomic leaf DNA samples used in the Southern blot in Figure 5 were analyzed, and twenty to twenty-four amplicons were sequenced from each plant line. This analysis revealed that cytosine residues in U3, the promoter region of *LORE1*a containing the three *Alu* I sites, are frequently methylated in control Gifu DNA, especially at CG and CHG sites (Blue bars in Figure 6A–6E). Graphical representation of the methylation status obtained from twenty amplicons showed some heterogeneity in the cytosine methylation patterns of the control Gifu (Figure S5A). This correlates with data obtained from the *Hin*d III and *Alu* I digestion patterns (Figure 5 and Figure S4). *LORE1*a is activated in plant no. 30, and compared with control Gifu, this line showed a dramatic decrease in the cytosine methylation level throughout the investigated region (Figure 6C and Figure S5D). Plant no. 3 possesses activated *LORE1*a and it showed a general decrease in the methylation level in the U3 region; in three of twenty-three amplicons a complete loss of cytosine methylation in U3 was observed (Figure 6A and Figure S5B). *LORE1*a remains inactive in plant no. 11, and methylation at CG and CHG sites was maintained, as well as being very evident in the U3 (Figure 6B and Figure S5C). Plant nos. 42 and 45 showed similar methylation patterns when averaged among clones (Figure 6D and 6E). However, two amplicons corresponding to alleles that were completely demethylated in U3 were observed in plant no. 45, in which *LORE1* is active, but not in no. 42, in which *LORE1* remains inactive (Figure S5E and S5F). Among the T0 plants analyzed, these data support the idea that there may be a correlation between *LORE1*a activation and the presence of *LORE1*a alleles that have totally lost cytosine methylation in U3.

To determine whether alteration in the methylation pattern occurs in the same region of other *LORE1* loci, we used bisulfite sequencing to determine the methylation status of two *LORE1* loci, *LORE1*b and *LORE1*f, which contain 5′ LTRs identical to that of *LORE1*a. This analysis revealed that the cytosine methylation profile of *LORE1*f is similar to that observed for *LORE1*a in control Gifu (blue bars in Figure 6K–6O). Specifically, it shows a higher level of methylation in the U3 region compared with the remaining regions in the investigated areas. However, in contrast to *LORE1*a, the methylation profile of *LORE1*f was largely unchanged among the five T0 plants investigated (red bars in Figure 6K–6O). *LORE1*b showed a moderate level of methylation throughout the investigated region in control Gifu, resulting in a flatter profile of methylation compared with *LORE1*a and *LORE1*f (blue bars in Figure 6F–6J). The significant decrease in methylation levels in *LORE1*b was observed in all the 5 T0 plants, even though the level of decrease differed (red bars in Figure 6F–6J). Taken together, the bisulfite sequencing unveiled variation of epigenetic status at *LORE1* loci in control Gifu plants and indicated alteration of this status in the five T0 plants investigated. A characteristic observed with *LORE1*a was the variability of epigenetic changes among the T0 plants, whereas *LORE1*b and *LORE1*f exhibited stability or rather similar changes among the five T0 plants.

## Discussion

In this study, we found that transposition of *LORE1*a, one of the *LORE1* elements present in the *Lotus japonicus* accession Gifu, can be activated in plants regenerated from de-differentiated cells. In addition, we show that *LORE1*a transposes in the male germline, giving rise to independent insertions in the progeny. The frequency of activation differs between populations (Table 1), but was independent of construct or antibiotics used to select transgenes. Combining all the data, we infer that the phenomenon observed here is a result of a series of processes. The first is a tissue culture step that induces epigenetic changes in *LORE1*a. This alteration was documented by observing variation in the cytosine methylation patterns among the T0 plants investigated. In turn, this variation leads to the preferential transposition of de-repressed *LORE1*a in the pollen of intact regenerated plants, since the *LORE1*a LTR promoter is specifically active in pollen grains. Finally, newly transposed copies in the male germlines are inherited by the following generation. Our data suggests that mechanisms regulating the tissue-specific activity of TEs should be taken into account when considering the biology of TEs and their impact on genome dynamics and evolution. Activation of *LORE1*a appears to be an attractive system for investigating these mechanisms, as well as for the experimental analysis of plant chromovirus behavior.

Once de-repressed, the transpositional activity of *LORE1*a was maintained for at least two generations, indicating that the retrotransposon escaped the re-establishment of silencing during this period. One possible explanation for this escape from silencing is the low level of transcription of *LORE1* in somatic cells, where *de novo* transcriptional silencing can be induced by an RNA-directed DNA methylation pathway. It has been shown that transcriptional gene silencing is inducible by artificial RNAi constructs utilizing the 35S promoter to drive the transgenes [36]. This promoter has been shown to be active in somatic tissues but not in pollen [37], and we do not know if *de novo* transcriptional silencing is inducible in pollen. Addition to that, it has been demonstrated that the RNA-directed transcriptional gene silencing and DNA methylation is less effective when the targets are located in the genic sequences, compared to those in the repetitive sequences [38]. Since *LORE1*a is located in an intron of a MAP kinase gene [28], efficiency of establishment of transcriptional gene silencing on once activated *LORE1*a may be low. An alternative possibility is that the increase in *LORE1* copy-number was insufficient to induce copy number-dependent silencing [33],[34]. However, re-silencing of *LORE1* was observed in the *nup133-3* mutant, which also has a low *LORE1* copy number. Interestingly, it was recently shown that pollen sperm cells accumulate transcripts of a set of genes involved in small RNA and DNA methylation pathways [39]. Furthermore, small RNAs of TEs originating from vegetative nuclei can transfer to sperm cells [40]. Investigation of the re-silencing of once-activated *LORE1*a in pollen, together with the steady state silencing of *LORE1*a, should provide new insights into the significance of epigenetic regulation in plant gametes.

Genetic changes, such as transposition of TEs and nucleotide substitutions and deletions generated during tissue culture, have been regarded as causes of the so-called somaclonal variations often observed as phenotypic changes in regenerated plant populations. Our investigation has unveiled another hidden layer of genetic changes creating phenotypic variation in regenerated plants. Epigenetic derepression of TEs induced via tissue culture can result in TE transpositions not in cultured cells but in regenerated plants. A similar behavior was observed for *Karma*, a rice LINE retrotransposon [41], and even though the underlying mechanism remains unclear, this observation indicates conservation of the feature. There are most likely other examples, but the temporal and spatial gaps between derepression and transposition of such TEs might have limited their detection. Our observation also indicates the potential use of tissue culture as a breeding method for generating epialleles of a gene of interest, even though these epialleles may not always be epigenetically stable, as demonstrated by recombinant inbred lines with epigenetically mosaic chromosomes consisting of wild-type and CG methylation-depleted segments [42]. Since epigenetic changes can be also generated in animal cells in culture [43], and considering the growing importance of the generative therapy using cultured stem cells, the risk of transposition of TEs after the regeneration of tissues should be given more attention and properly validated.

Even though we observed a good correlation between *LORE1*a activation and the presence of alleles with complete demethylation in the U3 of T0 plants, the presence of one amplicon of highly hypomethylated U3 in the control Gifu plant (completely hypomethylated except for one CG site, Figure S5A) suggests that demethylation alone might not be sufficient for *LORE1*a derepression. Therefore, there may be additional factors contributing to loss of *LORE1*a silencing in regenerated plants, but not in the Gifu plants. Alternatively, the changes in cytosine methylation pattern observed here may represent a by-product accompanying changes in chromatin states, such as histone modifications, which directly trigger *LORE1*a activation. Since the T0 plants analyzed in this study were selected with antibiotics during tissue culture, they are most likely of unicellular origin. Therefore, we suspect that the epigenetic variation at *LORE1*a that we observed among regenerated plants might already exist in cultured cells. Corroborating this suggestion is the finding that the epigenetic status of long-term cell cultures of *Arabidopsis* deviates from that of intact plants [44]. The range of epigenetic variation represented by the cytosine methylation pattern on *LORE1*a was more pronounced than in the other two *LORE1* loci investigated in T0 plants. This suggests that although different members of a TE family may possess over 99% identity, their epigenetic regulation may differ and that tissue culture could influence the silencing variably. Position effects might represent a possible explanation for the different epigenetic changes among the three loci. Potential position effects have been observed in maize, which shows low heritability of silencing of a *MuDR* element induced by the *Muk* locus, a *MuDR* derivative producing a hairpin RNA molecule [45]. The transcriptional regulation of the neighboring MAP kinase gene might also affect expression of *LORE1*a. Although *LORE1*b was dramatically demethylated in regenerated plants, we have not yet observed transcriptional or transpositional activation of the copy. This finding indicates that silencing of *LORE1*b may be achieved by methods other than DNA methylation, such as histone modification, or even non-epigenetically via mechanisms influenced by the surrounding sequence, as demonstrated by Cheng *et al.* [46].

Chromodomains of chromoviruses are categorized into three groups, according to their structural features. Reina, Tekay, and Galadriel chromodomains are classified into group II, while the *MAGGY* chromodomain belongs to group I [18],[19]. Group I chromodomains contain three conserved aromatic residues that are necessary for interaction with methylated H3K9. Group II chromodomains only retain the second of these residues. Since CRM chromodomains differ more than those of groups I and II, they are referred to as CR motifs [18]. Even though neither group II chromodomains nor CR motifs interact with histone H3 methyl-K9, the interacting partner of group I chromodomains, they are able to target a YFP fusion to heterochromatic regions when expressed in plant cells, suggesting that they interact with an unknown partner present in plant heterochromatin [18]. Following the standard classification, the *LORE1* and *LORE2* chromodomains both belong to group II (Figure S6). Although the group II chromodomain in *LORE1* appears canonical, we have not observed any strong global preference for insertion of *LORE1* into heterochromatin. However, since the *Lotus* genome project was focused on euchromatic regions [13], we cannot exclude the possibility that *LORE1* exhibits an insertional preference for gene-poor regions at a local level. In rice chromosome 1, the distribution pattern of chromoviruses possessing group II chromodomains suggest such a preference [18]. Future characterization of large numbers of new *LORE1*a insertion sites will, therefore, provide an opportunity to understand the biological function of the group II chromodomains. Gorinsek *et al*. pointed out that the genome of *L. japonicus* seems to contain a larger diversity of particular chromoviral clades than other plant species including *Medicago truncatula*, another model legume [16]. This may suggest that the *L. japonicus* genome was formed under the influence of the very active chromoviruses. Information on new insertion sites of *LORE1*a will also be useful for elucidating the survival strategy of these successfully propagated chromoviruses and the impact they have had on the current structure of the *L. japonicus* genome. From a different perspective, it might be interesting to see if the insertion site preference of *LORE1* is affected by the chromatin structure in the pollen where it transposes, since the features of chromatin in plant sperm cells are distinct from somatic cells. Usually chromatin in pollen sperm cells is transcriptionally active at the same time as being highly condensed; it may use sperm-specific variants of histone H3.3, which is a hallmark of active chromatin [47],[48].

It is possible that in bisexual flowering plants, TEs like *LORE1*, which are active in germlines, could be strong generators of genetic variation over a short evolutionary period. Furthermore, the uniparental activity of these TEs, i.e., showing transposition mainly in male gametophytes, might provide an advantage as a survival strategy. Activity in pollen minimizes the risk of adversely affecting fertility because the number of pollen grains is usually large. Since particular TE families often show distinct biases for one of the two sex chromosomes, uniparentally-active TEs might also be involved in formation of sex chromosomes, which are evolutionarily recent events in flowering plants [49],[50]. On a shorter time-scale, as in the transpositional activity of *LORE1*, gametophytic transposition, as well as the lack of strong bias for insertion sites and frequent insertions into genes, indicates that this retrotransposon could be an ideal tool for establishing an insertional gene tagging system. We estimate that the population size necessary to obtain at least one insertion allele for all genes at a 95% probability is approximately 200,000 plant lines in *L. japonicus*. This calculation is based on the following assumptions: the value 2.7, the highest average number of new copies observed here in a T1 plant derived from a T0 plant; 2.9 kb as the average gene size; and 472 Mb as the genome size of *L. japonicus* [13]. As *L. japonicus* is a perennial plant and can be propagated by cuttings, harvesting 200,000 seeds from the identified plants possessing active *LORE1* is feasible. We have started to establish a small-sized tagging population to test the system. Other transposable elements, activated in the same way as *LORE1*, might be identified in the course of establishment of this population; *LORE2* [29] is one such candidate.

### Note added during the production process

After the submission of this article, Tsukahara *et al.* reported the identification of a *Gypsy* element transposed in intact *ddm1* mutant plants of *Arabidopsis thaliana* [51]. Precise characterization of the behavior of the *Gypsy* element, together with that of *LORE1*, will facilitate our understanding of the interaction between LTR retrotransposons and plant genomes.

## Materials and Methods

### Plant materials

The Gifu accession of *Lotus japonicus* was used to generate both the transgenic and regenerated populations. The MG20 accession was used in the reciprocal crosspollination experiment with the T0 plant exhibiting *LORE1* activity. For promoter analysis of *LORE1* LTR using *Arabidopsis thaliana*, the ecotype Columbia was used to generate transgenic plants.

### Tissue culture methods

Transgenic and regenerated plant populations were produced from the Gifu accession using two different protocols. Populations 1 and 2 were generated according to the method described in [52]. Populations 3, 4, and 5 were generated following the method described in [53]. Antibiotic selection was not used when populations 2 and 5 were produced.

### Transformation of *Arabidopsis* and *L. japonicus* with the *LORE1* LTR::GUS fusion construct

The 225 bp LTR of *LORE1*a, corresponding to the region from 137 bp to 361 bp of the AJ966990 sequence, was cloned into a multi-cloning site upstream of an intron-containing GUS gene in the binary vector pZN-GUS [54]. The resulting plasmid was introduced into *Agrobacterium tumefaciens* strain EHA105. *Arabidopsis thaliana* ecotype Colombia was subsequently infected to generate transgenic plants following the method described in [55]. *L. japonicus* Gifu accession was infected with the same *Agrobacterium* strain and transgenic plants were generated following the method described in [53].

### DNA methods

Genomic Southern blots were carried out following the method described in [29]. *Hin*d III was used to digest genomic DNA in the Southern blot analyses shown in Figure 1 and Figure S2. *Hin*d III and *Alu* I were used to digest genomic DNA in Southern blot analyses shown in Figure 5 and Figure S4. Washes were performed at high stringency (65°C, 0.1x SSC, 0.1% SDS). The DNA probe used in Figure 1 and Figure S2 was generated by PCR using the primer pair *LORE1*gagF (5′-GTTGCCAGTATCGCCATGGACG-3′) and *LORE1*gagR (5′-GGATTGAGGCCTCCAAGATAAC-3′), and BAC DNA containing *LORE1*a [28]. The DNA probe used in Figure 5 and Figure S4 was generated by PCR using the primer pair 5′FLKF (5′-TTGACCTGCTCTTCAGTGCATG-3′) and 5′FLKR (5′-GAATCCGGGTATAAGGGTTCC-3′). The Megaprime DNA Labeling System (GE Healthcare) was used for labeling the DNA probes with alpha-^32^P-dCTP.

SSAP analyses to detect new *LORE1* insertions were conducted as described in [28]. In brief, genomic DNA was digested with *Mse* I (New England Biolabs), and ligated with *Mse* I adapters. The first PCR was conducted using a primer annealing to a internal region of *LORE1* and oriented outward, and a primer specific to the *Mse* I adapters. A nested PCR was conducted using the first PCR reaction as template. The amplified SSAP fragments were electrophoresed on polyacrylamide sequencing gels, and detected by silver staining. Bands for putative new insertions, i.e., absent from control Gifu analyses, were excised using a scalpel, boiled in 1x PCR buffer, and then used as a template to reamplify the fragment using the same primer pairs as in the nested PCR of the SSAP reaction. The reamplified fragments were electrophoresed on 1% agarose gels, excised, and extracted from the gel using Wizard SV Gel and PCR Clean-up System (Promega). Cleaned fragments were sequenced using a BigDye Terminator v3.1 Cycle Sequencing Kit (Applied Biosystems). The reamplified fragments would be expected to contain the junction sequence between *LORE1* and its flanking DNA, in which *Mse* I sites are absent. Sequences that contained *Mse* I sites were regarded as artifacts and not subjected to further analyses. To amplify junctions between the flanking DNA and *LORE1*, we designed primers specific to the flanking sequences obtained and oriented toward *LORE1*. When genome sequences corresponding to flanking DNA were available on the database, they were utilized to design primers. We confirmed that amplifications were successful for plants from which the SSAP fragments were recovered, but not from the parent plant or control Gifu accession.

DNA sequences corresponding to regions 1 and 2 in newly transposed *LORE1* elements were obtained by direct sequencing of PCR products. These were amplified by primers specific to the 5′ flanking sequences of each *LORE1* element and primer 4 (5′-CAACAGTAGTATCAAATGTAGG-3′), as indicated in Figure 1A, using a BigDye Terminator v3.1 Cycle Sequencing Kit. The primers used for sequencing region 1 were Reg1F (5′-AGTAGCACCTGTAACAGTGGAG-3′) and Reg1R (5′-CATTAAGAGAGACTTTAGGAAC-3′), and those for region 2 were Reg2F (5′-CCTCCAACATTGTCAGTGATAG-3′) and Reg2R (5′-TAGCTGTAAAGCTCCTGTCCAC-3′). In the reciprocal cross analysis shown in Figure 1D, PCR reactions were performed using Primer 1 (5′-GACTAAGTGCCTCTTCAACTGC-3′) and Primer 2 (5′-GACTAAGTGCCTCTTCAACTGC-3′) to amplify *LORE1*a from Gifu, and Primer 1 and Primer 3 (5′-CACCTGACGATGCTAGCCTTGG-3′) to amplify the region allelic to *LORE1*a (absence of *LORE1*) from MG20 (see Figure 1 legend).

### Bisulfite sequencing

Genomic DNA samples were extracted from the leaves of T0 plants. Sodium bisulfite treatment of the DNA was conducted using a BisulFast Methylated DNA Detection Kit (TOYOBO), following the manufacturer's instructions. Briefly, 1 µg of column-purified genomic DNA was digested with *Eco* RI, treated with Proteinase K, and then subjected to bisulfite modification. Bisulfite-treated DNA (1 µl) was used as template for PCR reactions. Primary and nested PCR reactions were conducted for each *LORE1* locus. The following primers were used for the primary PCR reactions: BSF R1 (5′-CTCTRAAACCTTRTTRCTTCARCCAT-3′) in combination with BSFa F (5′-TAAAAGAGAATYTGGGTATAAGGGAA-3′) for *LORE1*a; BSFb F (5′-TTYAAAGGTGYAGTYTYAATTGTATT-3′) for *LORE1*b; and BSFf F (5′-AGGGAGAYGAYAGTGATGGTGTTTT-3′) for *LORE1*f. For nested PCR reactions, 1 µl of the primary PCR reaction was used as template, with the following primers: for *LORE1*a, BSF R2 (5′- CCATRATTCRCTCCTCCRCTTCAC-3′) and BSFa F; for *LORE1*b, BSF R2 and BSFb F; and for *LORE1*f, BSF R2 and BSFf F. PCR reactions (20 µl) were conducted as follows: incubation at 94°C for 2 min as an initial denaturation step, 30 cycles of 30 s at 94°C, 45 s at 55°C, and 45 s at 72°C for amplification, and incubation at 72°C for 5 min. Amplified fragments were TA cloned using the pGEM-T Easy Vector System (Promega). For *LORE1*a, 6 to 8 TA clones were obtained from each of three PCR reactions and, in total, between 20 and 24 clones were sequenced for each plant analyzed. For *LORE1*b, 12 clones obtained from a PCR reaction were analyzed for each plant examined. For *LORE1*f, 11 or 12 clones obtained from a PCR reaction were analyzed for each plant examined.

### Total RNA extraction and RT–PCR

A method modified from [56] was used for RNA isolation from plant tissues. Ground tissues (∼0.1 g) were incubated with 700 µl of extraction buffer (2% ß-mercaptoethanol, 2% hexadecyltrimethylammonium bromide, 100 mM Tris-HCl [pH 8.0], and 25 mM EDTA) at room temperature for less than 5 min. The recovered RNA was treated with 5 U DNase I at 37°C for 30 min in a 100 µl reaction. DNase-treated RNA was purified and recovered using an RNeasy Mini Kit (QIAGEN), with additional DNase treatment performed on a column, following the manufacturer's instructions. For RT-PCR, cDNA was synthesized by ReverTra Ace α (TOYOBO) using 1 µg of purified total RNA and oligo (dT) 20 primer in a 20 µl reaction. A 5× dilution of the cDNA reaction (2 µl) was used as template for semi-quantitative RT-PCR in a 20 µl PCR reaction using Ex Taq (TaKaRa) and 5 pmoles of each primer. The primers *LORE1*gagF and *LORE1*gagR were used for detection of *LORE1* transcripts and products were amplified with 28 PCR cycles. As a control, the primers EF1αF (5′-GTGAGGGACATGAGACAGACTG-3′) and EF1αR (5′-AAATAGCAGTGTAGGACAAGTC-3′) were used for detection of transcripts of elongation factor 1 alpha, and these reactions required 24 PCR amplification cycles. To identify the transcription of *LORE1* members, RT-PCR amplifications of regions 1 and 2 were conducted using the primer pairs Reg1 F and Reg1 R, or Reg2 F and Reg2 R, respectively.

### Sequence data analysis

BLAST searches were used to identify sequences homologous to SSAP fragments. These were conducted using Miyakogusa jp (http://www.kazusa.or.jp/lotus/), NCBI BLAST (http://blast.ncbi.nlm.nih.gov/Blast.cgi), and Phytozome *Glycine max* (http://www.phytozome.net/soybean). Pfam was accessed at http://pfam.sanger.ac.uk/. Bisulfite sequencing data was analyzed using QUMA [57] and CyMATE [58].

## Supporting Information

Figure S1 SSAP analysis for detecting new *LORE1* insertions. T0 and T1 plants used in Figure 1B were analyzed by 5′ and 3′ SSAP to detect new *LORE1* insertions. Bands marked with red asterisks were confirmed by PCR to have originated from new insertions in the T1 plant.
(0.66 MB TIF)

Figure S2 *LORE1* activity over generations. The results from Southern blots of genomic DNA digested with *Hin*d III and hybridized with the probe indicated in Figure 1A are shown. (A) Continuing *LORE1* transposition in T1 plants that already possess an increased number of *LORE1* elements. *LORE1* copy number was analyzed by genomic Southern blot analysis in one T1 plant and five T2 progeny from each of three plant lines (A–C). New bands were detected in T2 progeny, suggesting that *LORE1*is still active in T1. (B) *LORE1* is inactivated in the *nup133-3* mutant line. Genomic Southern blot detected an additional band in the mutant plants; however, the absence of polymorphic bands among the *nup133-3* plants indicates no transposition after the initial activation giving rise to the *nup133-3* allele. These data indicate that *LORE1* has been repressed, at least in the progeny analyzed.
(1.24 MB TIF)

Figure S3 Promoter activity of the *LORE1*a LTR is demonstrated in *Arabidopsis*. Histochemical analysis of GUS expression in *Arabidopsis* plants transformed with a *LORE1*a LTR::GUS fusion. (A) Inflorescence assayed for 48 h. The long incubation revealed that the LTR exhibits promoter activity in the young developing anthers of flower buds marked with black arrowheads. GUS activity in the pollinated flower, marked with a white arrowhead, was more visible after this prolonged incubation than after the 12 h incubation shown in Figure 5F. (B) Close-up of the ovary of the pollinated flower marked by the white arrowhead in (A). Blue stained pollen tubes running to the ovules (O) and a bundle of pollen tubes in the transmitting tract were observed. (C and E) Dark field images of cross-sections of the youngest (C) and oldest (E) GUS-positive anthers shown in (A). Anthers were embedded in Technovit 7100 (Heraeus Kulzer) and sectioned. GUS activity was visualized as red signals. (D,F) DIC images of the same samples shown in (C,E), respectively. Higher GUS activity was detected in the surrounding cell layers than in young, developing pollen grains (pg), expected to be undergoing meiosis (C,D) and mitosis I (E,F) stages, respectively.
(2.44 MB TIF)

Figure S4 Cytosine methylation status at three *Alu* I sites in the 5′ LTR of *LORE1*a is compared between leaves and flowers. Genomic Southern blot detected fragments containing 5′ flanking DNA from *LORE1*a. DNA samples of control Gifu and four T0 plants (nos. 3, 11, 30, and 45), extracted from leaves (left) and flowers (right) respectively, were digested with *Hin*d III alone or double digested with *Hin*d III and *Alu* I. Plants marked with + show transpositional activity of *LORE1* and those marked with - do not. Molecular sizes of the DNA makers and the bands detected are indicated on the left. The banding patterns observed in leaves and flowers were consistent with each other.
(0.38 MB TIF)

Figure S5 Cytosine methylation statuses of the 5′ LTR and its surrounding region in *LORE1*a of control Gifu and five T0 plants (nos. 3, 11, 30, 42, and 45) are predicted from bisulfite PCR amplicons using CyMATE [58]. Red circles: cytosine residues in a CG context, Blue rectangles: cytosine residues in a CHG context, Green triangles: cytosine residues in a CHH context. Filled symbols indicate methylated sites and open symbols indicate demethylated sites. Asterisks indicate the three CHG sites present in the three *Alu* I sites used to assay the cytosine methylation status in the Southern blot analysis shown in Figure 5 and Figure S4.
(2.46 MB TIF)

Figure S6 Alignment of chromodomains. Chromodomains of two cellular proteins and five chromoviruses were aligned using CLUSTAL W (available at the DDBJ web site: http://clustalw.ddbj.nig.ac.jp/top-j.html). Arrowheads indicate the three amino acid residues in the chromodomain of HP1a that interact with methylated lysine residues on histone H3; these are highly conserved in authentic cellular chromodomains and group I chromodomains in chromoviruses [18],[19]. Chromodomain sequences in *LORE1* and *LORE2* were predicted using Pfam (http://pfam.sanger.ac.uk/) based on their nucleotide sequences. These chromodomains are also classified in group II, according to previous work [18],[19]. Other sequences were obtained from [18]. Dm HP1a, *Drosophila melanogaster* HP1a; AT LHP1∶TFL2, *Arabidopsis thaliana* Terminal Flower 2; Mg MAGGY, *Magnaporthe oryzae* MAGGY; Lj LORE2, *Lotus japonicus LORE2*; Os CHDII, *Oryza sativa* RIRE3-like element; Lj LORE1a, *Lotus japonicus LORE1*a; At Tma, *Arabidopsis thaliana* TMA.
(0.33 MB TIF)
