# Supplementary figures and images for: Derepression of the Plant Chromovirus LORE1 Induces Germline Transposition in Regenerated Plants
Source: PLoS Genet. 2010 Mar 5;6(3):e1000868. doi: 10.1371/journal.pgen.1000868 (PMC2832683; doi:10.1371/journal.pgen.1000868)

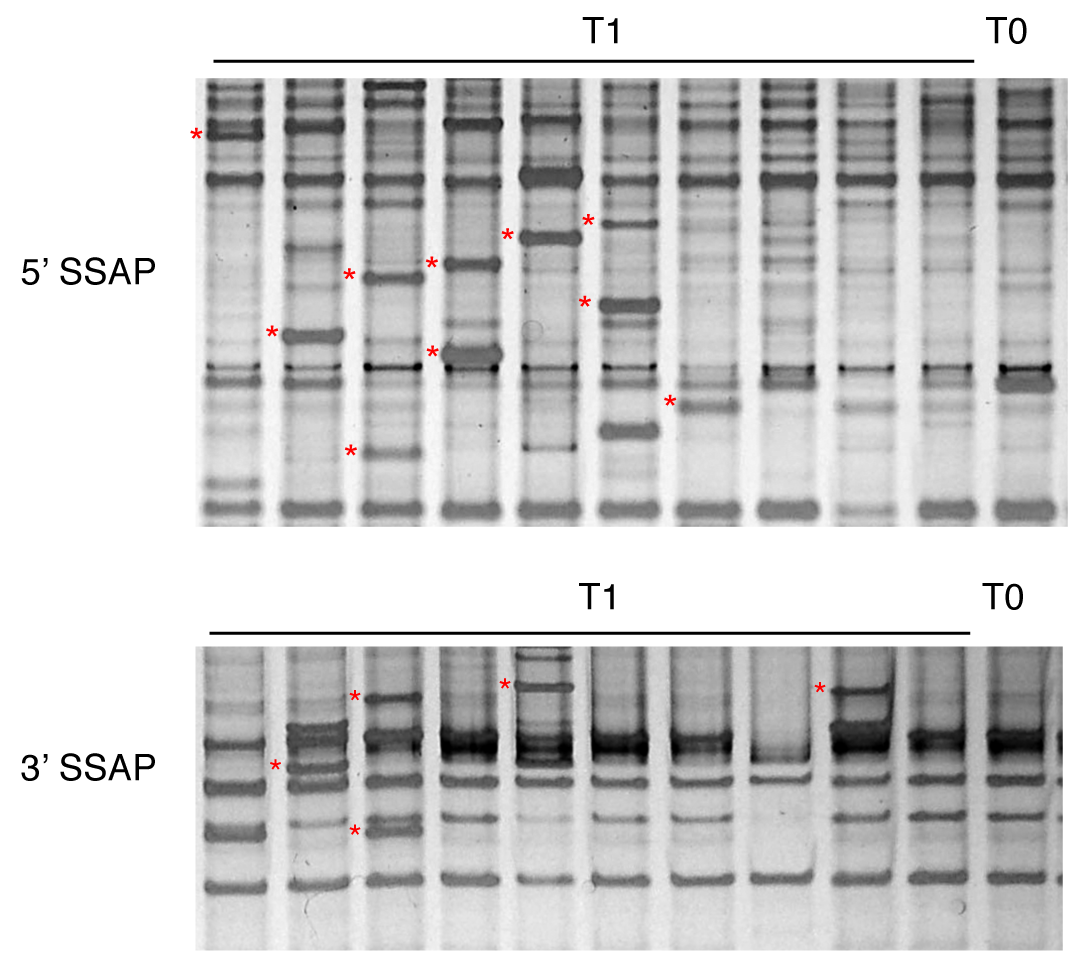

Supplement: Figure S1 — SSAP analysis for detecting new LORE1 insertions. T0 and T1 plants used in Figure 1B were analyzed by 5′ and 3′ SSAP to detect new LORE1 insertions. Bands marked with red asterisks were confirmed by PCR to have originated from new insertions in the T1 plant. (0.66 MB TIF) [file pgen.1000868.s001.tif]

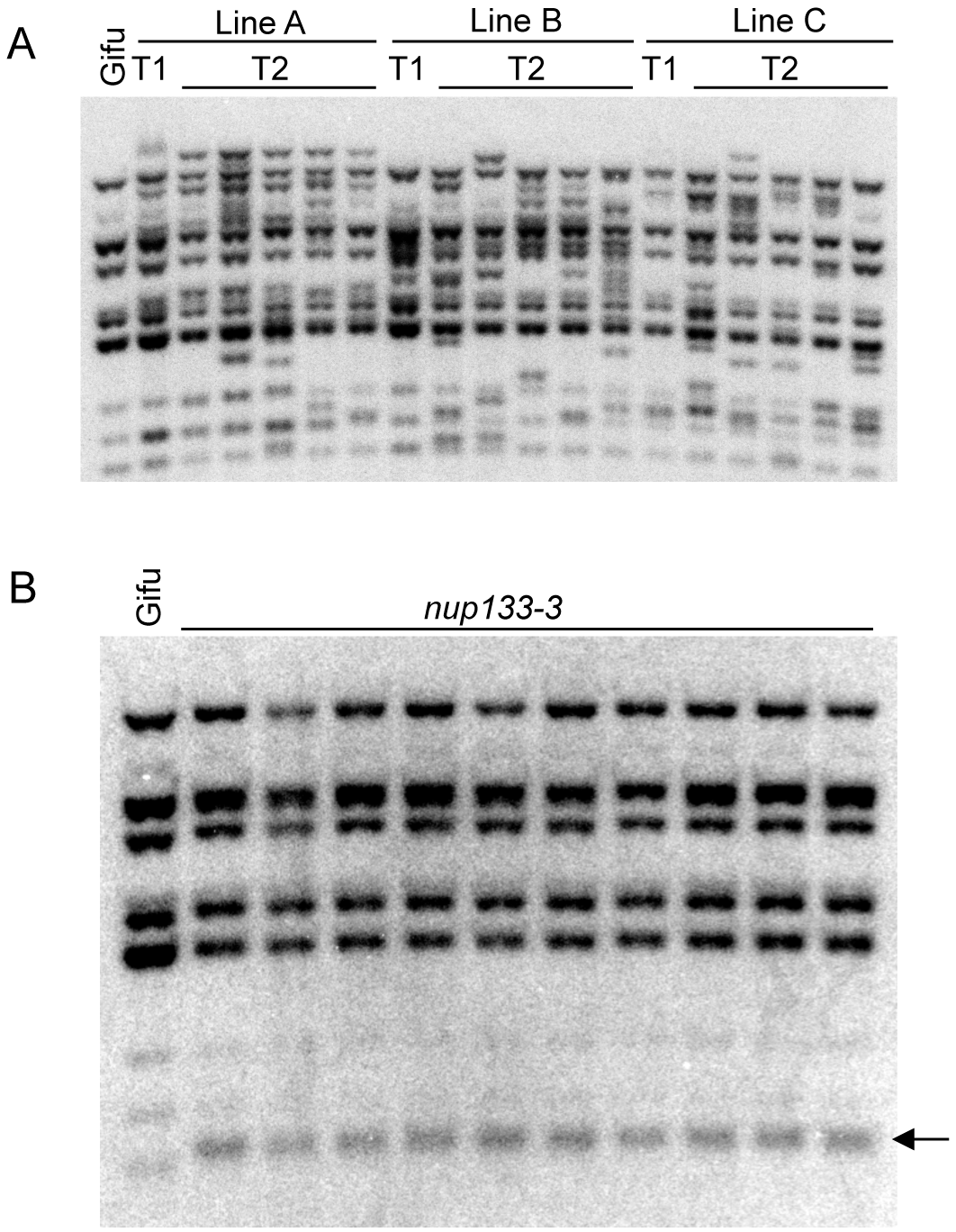

Supplement: Figure S2 — LORE1 activity over generations. The results from Southern blots of genomic DNA digested with Hind III and hybridized with the probe indicated in Figure 1A are shown. (A) Continuing LORE1 transposition in T1 plants that already possess an increased number of LORE1 elements. LORE1 copy number was analyzed by genomic Southern blot analysis in one T1 plant and five T2 progeny from each of three plant lines (A–C). New bands were detected in T2 progeny, suggesting that LORE1is still active in T1. (B) LORE1 is inactivated in the nup133-3 mutant line. Genomic Southern blot detected an additional band in the mutant plants; however, the absence of polymorphic bands among the nup133-3 plants indicates no transposition after the initial activation giving rise to the nup133-3 allele. These data indicate that LORE1 has been repressed, at least in the progeny analyzed. (1.24 MB TIF) [file pgen.1000868.s002.tif]

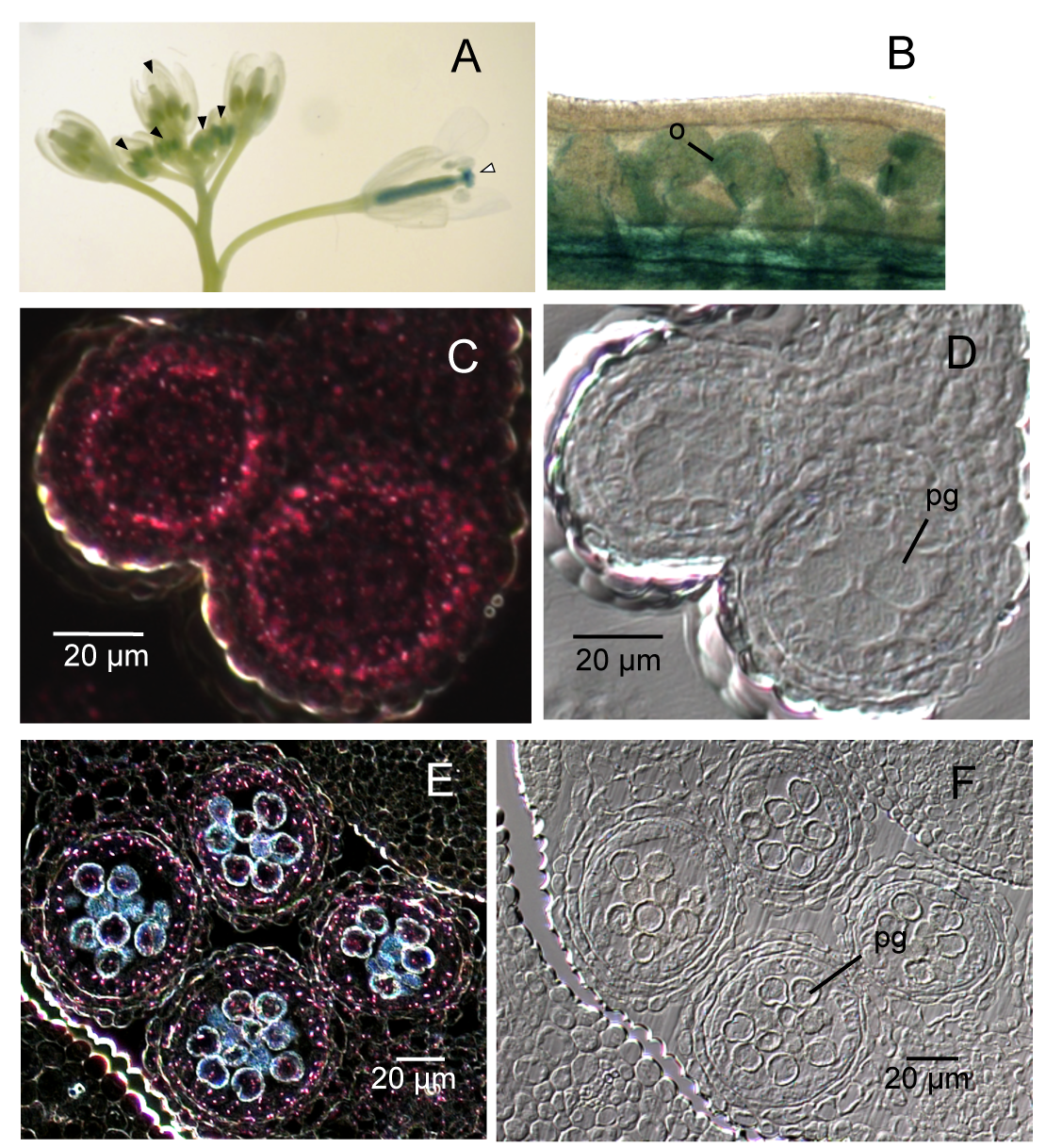

Supplement: Figure S3 — Promoter activity of the LORE1a LTR is demonstrated in Arabidopsis. Histochemical analysis of GUS expression in Arabidopsis plants transformed with a LORE1a LTR::GUS fusion. (A) Inflorescence assayed for 48 h. The long incubation revealed that the LTR exhibits promoter activity in the young developing anthers of flower buds marked with black arrowheads. GUS activity in the pollinated flower, marked with a white arrowhead, was more visible after this prolonged incubation than after the 12 h incubation shown in Figure 5F. (B) Close-up of the ovary of the pollinated flower marked by the white arrowhead in (A). Blue stained pollen tubes running to the ovules (O) and a bundle of pollen tubes in the transmitting tract were observed. (C and E) Dark field images of cross-sections of the youngest (C) and oldest (E) GUS-positive anthers shown in (A). Anthers were embedded in Technovit 7100 (Heraeus Kulzer) and sectioned. GUS activity was visualized as red signals. (D,F) DIC images of the same samples shown in (C,E), respectively. Higher GUS activity was detected in the surrounding cell layers than in young, developing pollen grains (pg), expected to be undergoing meiosis (C,D) and mitosis I (E,F) stages, respectively. (2.44 MB TIF) [file pgen.1000868.s003.tif]

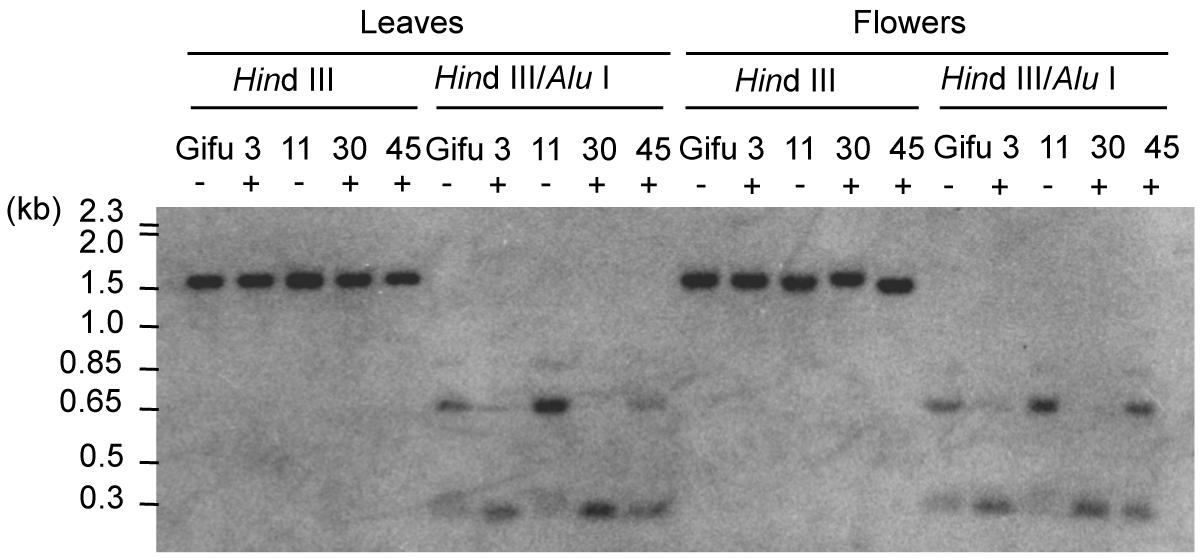

Supplement: Figure S4 — Cytosine methylation status at three Alu I sites in the 5′ LTR of LORE1a is compared between leaves and flowers. Genomic Southern blot detected fragments containing 5′ flanking DNA from LORE1a. DNA samples of control Gifu and four T0 plants (nos. 3, 11, 30, and 45), extracted from leaves (left) and flowers (right) respectively, were digested with Hind III alone or double digested with Hind III and Alu I. Plants marked with + show transpositional activity of LORE1 and those marked with - do not. Molecular sizes of the DNA makers and the bands detected are indicated on the left. The banding patterns observed in leaves and flowers were consistent with each other. (0.38 MB TIF) [file pgen.1000868.s004.tif]

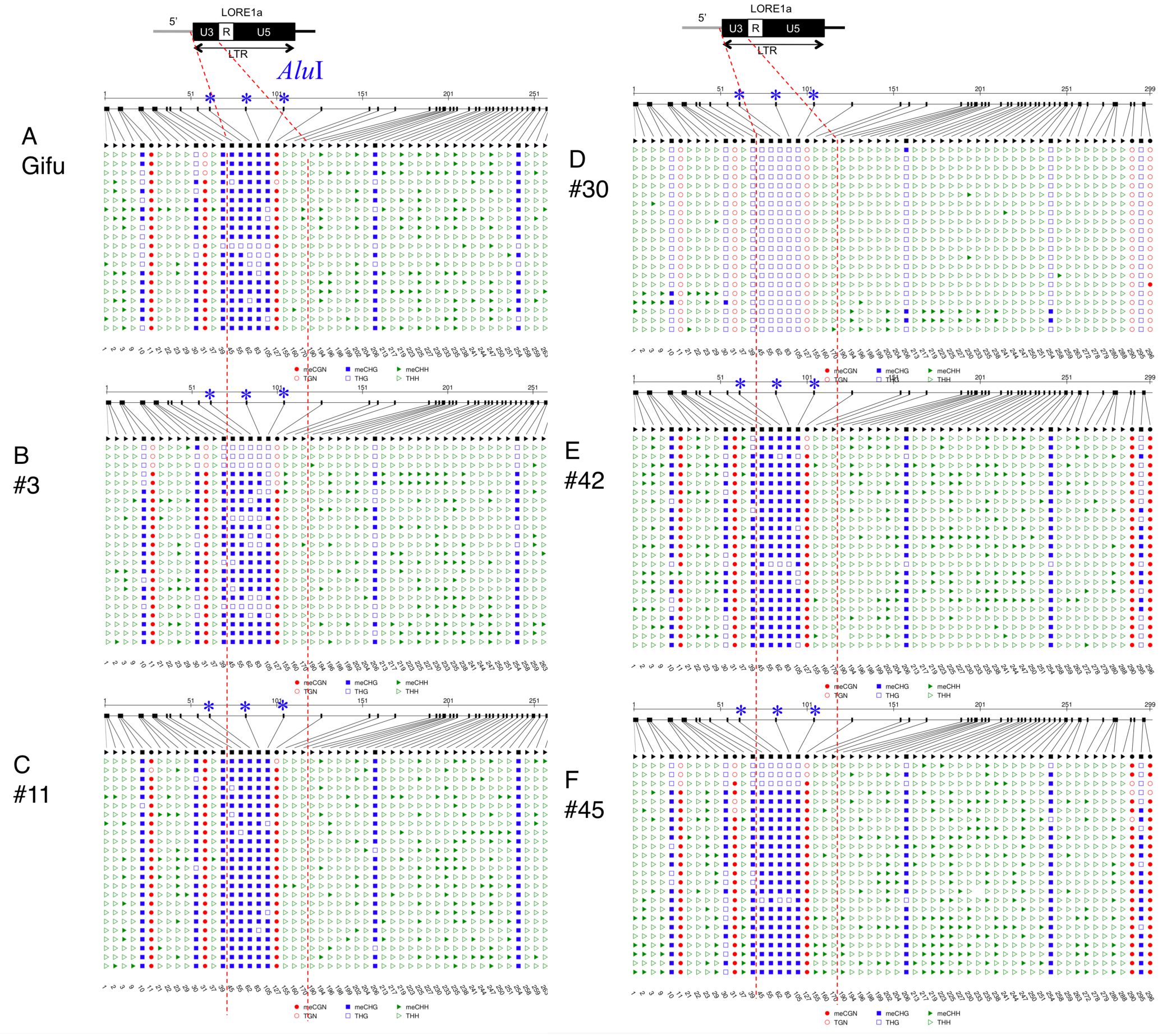

Supplement: Figure S5 — Cytosine methylation statuses of the 5′ LTR and its surrounding region in LORE1a of control Gifu and five T0 plants (nos. 3, 11, 30, 42, and 45) are predicted from bisulfite PCR amplicons using CyMATE [58]. Red circles: cytosine residues in a CG context, Blue rectangles: cytosine residues in a CHG context, Green triangles: cytosine residues in a CHH context. Filled symbols indicate methylated sites and open symbols indicate demethylated sites. Asterisks indicate the three CHG sites present in the three Alu I sites used to assay the cytosine methylation status in the Southern blot analysis shown in Figure 5 and Figure S4. (2.46 MB TIF) [file pgen.1000868.s005.tif]

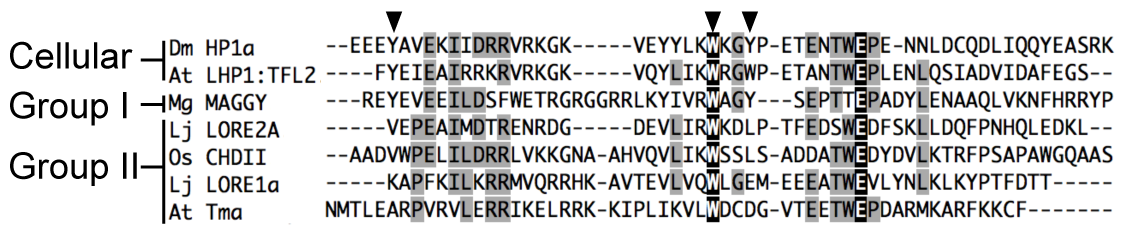

Supplement: Figure S6 — Alignment of chromodomains. Chromodomains of two cellular proteins and five chromoviruses were aligned using CLUSTAL W (available at the DDBJ web site: http://clustalw.ddbj.nig.ac.jp/top-j.html). Arrowheads indicate the three amino acid residues in the chromodomain of HP1a that interact with methylated lysine residues on histone H3; these are highly conserved in authentic cellular chromodomains and group I chromodomains in chromoviruses [18],[19]. Chromodomain sequences in LORE1 and LORE2 were predicted using Pfam (http://pfam.sanger.ac.uk/) based on their nucleotide sequences. These chromodomains are also classified in group II, according to previous work [18],[19]. Other sequences were obtained from [18]. Dm HP1a, Drosophila melanogaster HP1a; AT LHP1∶TFL2, Arabidopsis thaliana Terminal Flower 2; Mg MAGGY, Magnaporthe oryzae MAGGY; Lj LORE2, Lotus japonicus LORE2; Os CHDII, Oryza sativa RIRE3-like element; Lj LORE1a, Lotus japonicus LORE1a; At Tma, Arabidopsis thaliana TMA. (0.33 MB TIF) [file pgen.1000868.s006.tif]
